# Evaluation of Inequities in Cancer Treatment Delay or Discontinuation Following SARS-CoV-2 Infection

**DOI:** 10.1001/jamanetworkopen.2022.51165

**Published:** 2023-01-13

**Authors:** Adana A. M. Llanos, Adiba Ashrafi, Nabarun Ghosh, Jennifer Tsui, Yong Lin, Angela J. Fong, Shridar Ganesan, Carolyn J. Heckman

**Affiliations:** 1 Department of Epidemiology, Mailman School of Public Health, Columbia University Irving Medical Center, New York, New York; 2 Herbert Irving Comprehensive Cancer Center, Columbia University Irving Medical Center, New York, New York; 3 Department of Biostatistics and Epidemiology, Rutgers School of Public Health, Piscataway, New Jersey; 4 Keck School of Medicine, University of Southern California, Los Angeles; 5 Rutgers Cancer Institute of New Jersey, New Brunswick; 6 Department of Medicine, Rutgers Robert Wood Johnson Medical School, New Brunswick, New Jersey; 7 Department of Medicine and Pharmacology, Rutgers Robert Wood Johnson Medical School, New Brunswick, New Jersey

## Abstract

**Importance:**

There is a disproportionately greater burden of COVID-19 among Hispanic and non-Hispanic Black individuals, who also experience poorer cancer outcomes. Understanding individual-level and area-level factors contributing to inequities at the intersection of COVID-19 and cancer is critical.

**Objective:**

To evaluate associations of individual-level and area-level social determinants of health (SDOH) with delayed or discontinued cancer treatment following SARS-CoV-2 infection.

**Design, Setting, and Participants:**

This retrospective, registry-based cohort study used data from 4768 patients receiving cancer care who had positive test results for SARS-CoV-2 and were enrolled in the American Society for Clinical Oncology COVID-19 Registry. Data were collected from April 1, 2020, to September 26, 2022.

**Exposures:**

Race and ethnicity, sex, age, and area-level SDOH based on zip codes of residence at the time of cancer diagnosis.

**Main Outcomes and Measures:**

Delayed (≥14 days) or discontinued cancer treatment (any cancer treatment, surgery, pharmacotherapy, or radiotherapy) and time (in days) to restart pharmacotherapy.

**Results:**

A total of 4768 patients (2756 women [57.8%]; 1558 [32.7%] aged ≥70 years at diagnosis) were included in the analysis. There were 630 Hispanic (13.2%), 196 non-Hispanic Asian American or Pacific Islander (4.1%), 568 non-Hispanic Black (11.9%), and 3173 non-Hispanic White individuals (66.5%). Compared with non-Hispanic White individuals, Hispanic and non-Hispanic Black individuals were more likely to experience a delay of at least 14 days or discontinuation of any treatment and drug-based treatment; only estimates for non-Hispanic Black individuals were statistically significant, with correction for multiple comparisons (risk ratios [RRs], 1.35 [95% CI, 1.22-1.49] and 1.37 [95% CI, 1.23-1.52], respectively). Area-level SDOH (eg, geography, proportion of residents without health insurance or with only a high school education, lower median household income) were associated with delayed or discontinued treatment. In multivariable Cox proportinal hazards regression models, estimates suggested that Hispanic (hazard ratio [HR], 0.87 [95% CI, 0.71-1.05]), non-Hispanic Asian American or Pacific Islander (HR, 0.79 [95% CI, 0.46-1.35]), and non-Hispanic Black individuals (HR, 0.81 [95% CI, 0.67-0.97]) experienced longer delays to restarting pharmacotherapy compared with non-Hispanic White individuals.

**Conclusions and Relevance:**

The findings of this cohort study suggest that race and ethnicity and area-level SDOH were associated with delayed or discontinued cancer treatment and longer delays to the restart of drug-based therapies following SARS-CoV-2 infection. Such treatment delays could exacerbate persistent cancer survival inequities in the United States.

## Key Points

**Question:**

Among individuals with cancer, are members of racial and ethnic minority groups and those residing in socioeconomically disadvantaged areas more likely to experience cancer treatment delays and discontinuation after SARS-CoV-2 infection relative to non-Hispanic White individuals and those residing in areas of higher socioeconomic status?

**Findings:**

In this cohort study of 4768 patients with cancer, non-Hispanic Black and Hispanic individuals were more likely to have cancer treatment delays of at least 14 days or treatment discontinuation relative to non-Hispanic White individuals; there was evidence of associations between area-level social determinants of health with cancer treatment delay or discontinuation.

**Meaning:**

These findings suggest that COVID-19–related cancer treatment delays among vulnerable populations might contribute to worsening cancer survival inequities in the future.

## Teaser

This cohort study evaluates associations of patient-level sociodemographic and clinical factors and area-level social determinants of health with delayed or discontinued cancer treatment following a confirmed positive SARS-CoV-2 test result among patients with cancer.

## Introduction

The COVID-19 pandemic has disrupted oncology screening, diagnostic, and treatment practices.^1,2^ At least 3 major care delivery challenges arose from pandemic conditions. First, throughout the pandemic, oncology clinicians had to balance the risk of delaying appointments with patient exposure to SARS-CoV-2, the virus that causes COVID-19.^1,2^ This was especially difficult when case counts were surging (ie, at the start of the pandemic, before vaccine availability), as clinicians recognized increased susceptibility of patients with cancer to COVID-19–related complications and hospitalization.^3,4,5,6,7,8,9,10^ In addition, patients with cancer and comorbid conditions, such as cardiovascular disease, diabetes, and kidney disease, had a far greater risk of SARS-CoV-2 exposure due to the need for oncology care and chronic disease management.^11^ Second, physical distancing requirements limited clinicians’ ability to guarantee high-quality care and prevented patients’ caregivers from being present to provide practical and social support during clinical encounters.^1^ Finally, many health care facilities were forced to allocate numerous resources (ie, intensive care unit beds, ventilators, staffing) to patients with COVID-19, reducing their availability to cancer patients.^1^

Black and Hispanic communities shoulder a disproportionate burden of poor cancer outcomes^12,13^ and are inordinately impacted by COVID-19.^14,15,16,17^ These communities also disproportionately seek care in safety-net settings, which were more likely to be short-staffed or have limited resources throughout the pandemic.^18^ Since suboptimal and delayed cancer treatment are associated with poor survival, understanding the factors associated with COVID-19–related treatment delays among patients with cancer is crucial to understanding the pandemic’s long-term impact.

The objective of this study was to evaluate associations of patient-level factors (ie, sociodemographic and clinical factors) and area-level social determinants of health (SDOH) with delayed (by ≥14 days of scheduled treatment) or discontinued cancer treatment following a confirmed positive SARS-CoV-2 test result in a diverse cohort of patients with cancer. We hypothesized that members of racial and ethnic minority groups and individuals residing in socioeconomically disadvantaged areas would be more likely to experience cancer treatment delays and discontinuation compared with White individuals and those residing in higher socioeconomic status (SES) areas.

## Methods

### Study Design

This registry-based cohort study was conducted using data from the American Society for Clinical Oncology (ASCO) COVID-19 Registry^19^ in compliance with the Strengthening the Reporting of Observational Studies in Epidemiology (STROBE) reporting guideline for cohort studies. The Columbia University Irving Medical Center Institutional Review Board approved this study and waived the need for informed consent because the ASCO COVID-19 Registry was deemed nonhuman participant research by the Western Institutional Review Board.

### Data Source

Since April 2020, the ASCO COVID-19 Registry has collected information on COVID-19 treatment and outcomes and cancer treatment and outcomes for individuals with cancer in the US with a confirmed positive SARS-CoV-2 test result. Participating oncology practices—including private, hospital or health system, and academic practices—identified patients with cancer and a confirmed SARS-CoV-2 infection who were (1) newly diagnosed and undergoing cancer staging and/or receipt of initial treatment, (2) receiving anticancer treatment, (3) receiving supportive care, or (4) cancer-free for less than 12 months and receiving adjuvant (ie, hormonal) treatment. Data collection methods have been described in detail elsewhere.^19^

Data for the present study were collected from April 1, 2020, to September 26, 2022.The ASCO COVID-19 Registry added data from new and existing registry patients through July 31, 2022, which were received as a limited data set (N = 5262) in September 2022 for the current study. Individuals with missing or unknown race or ethnicity (n = 2), sex (n = 8), age at positive SARS-CoV-2 test result (n = 1), and/or vital status (n = 30) were excluded from the analysis. An additional 453 individuals were excluded due to our inability to access the date of death (ie, Health Insurance Portability and Accountability Act Safe-Harbor method for deidentification), resulting in an analytic sample of 4768 patients. Because race and ethnicity were hypothesized to be key factors of interest and there were far too few non-Hispanic American Indian or Alaska Native individuals (177 [3.7%]) and 24 individuals (0.5%) who selected the other ethnicity category (including 5 [0.1%] American Indian or Alaska Native, 8 [0.2%] Asian American or Pacific Islander, 5 [0.1%] Black, and 6 [0.1%] White patients), these groups were included in the descriptive analysis (Table 1) but excluded from the main analysis.

**Table 1.** Characteristics of Patients With Cancer in the American Society for Clinical Oncology COVID-19 Registry at Study Entry

Characteristic	Patient group, No. (%)^a^					
	Overall (N = 4768)^b^	Race and ethnicity				
		Hispanic (n = 630)	Non-Hispanic Asian American or Pacific Islander (n = 196)	Non-Hispanic Black (n = 568)	Non-Hispanic White (n = 3173)	*P* value^c^
**Sociodemographic**						
Sex						
Men	2012 (42.2)	257 (40.8)	80 (40.8)	217 (38.2)	1370 (43.2)	.13
Women	2756 (57.8)	373 (59.2)	116 (59.2)	351 (61.8)	1803 (56.8)	
Age at COVID-19 diagnosis, y						
<50	858 (18.0)	202 (32.1)	47 (24.0)	138 (24.3)	434 (13.7)	<.001
50-59	987 (20.7)	172 (27.3)	41 (20.9)	120 (21.1)	602 (19.0)	
60-69	1365 (28.6)	147 (23.3)	46 (23.5)	166 (29.2)	954 (30.1)	
≥70	1558 (32.7)	109 (17.3)	62 (31.6)	144 (25.4)	1183 (37.3)	
**Health history**						
BMI^d^						
<25	1316 (27.6)	159 (25.2)	70 (35.7)	148 (26.1)	878 (27.7)	.009
25-29	1474 (30.9)	190 (30.2)	61 (31.1)	151 (26.6)	1005 (31.7)	
30-34	903 (18.9)	121 (19.2)	30 (15.3)	106 (18.7)	611 (19.3)	
≥35	875 (18.4)	120 (19.0)	28 (14.3)	133 (23.4)	564 (17.8)	
History of tobacco use						
Current	416 (8.7)	34 (5.4)	14 (7.1)	66 (11.6)	289 (9.1)	<.001
Former	1804 (37.8)	173 (27.5)	74 (37.8)	191 (33.6)	1310 (41.3)	
Never	2394 (50.2)	407 (64.6)	96 (49.0)	292 (51.4)	1490 (47.0)	
Unsure	154 (3.2)	16 (2.5)	12 (6.1)	19 (3.3)	84 (2.6)	
No. of comorbidities requiring active treatment in the past 12 mo						
0	1799 (37.7)	282 (44.8)	75 (38.3)	137 (24.1)	1200 (37.8)	<.001
1	1385 (29.0)	140 (22.2)	48 (24.5)	189 (33.3)	954 (30.1)	
2	875 (18.4)	105 (16.7)	34 (17.3)	141 (24.8)	579 (18.2)	
≥3	610 (12.8)	55 (8.7)	24 (12.2)	95 (16.7)	412 (13.0)	
**Cancer status**						
Cancer type^e^						
Breast	1166 (24.5)	163 (25.9)	46 (23.5)	145 (25.5)	765 (24.1)	<.001
Gastrointestinal	725 (15.2)	144 (22.9)	34 (17.3)	80 (14.1)	436 (13.7)	
Gynecologic or genitourinary	681 (14.3)	93 (14.8)	33 (16.8)	100 (17.6)	425 (13.4)	
Lymphoid, hematopoietic, or related tissue	1094 (22.9)	116 (18.4)	40 (20.4)	122 (21.5)	780 (24.6)	
Respiratory or intrathoracic organ	519 (10.9)	23 (3.7)	23 (11.7)	61 (10.7)	383 (12.1)	
Other	583 (12.2)	91 (14.4)	20 (10.2)	60 (10.6)	384 (12.1)	
Solid tumor						
No	1233 (25.9)	149 (23.7)	50 (25.5)	151 (26.6)	844 (26.6)	.48
Yes	3535 (74.1)	481 (76.3)	146 (74.5)	417 (73.4)	2329 (73.4)	
Cancer stage^f^						
Local	1161 (24.3)	199 (31.6)	45 (23.0)	140 (24.6)	722 (22.8)	.01
Regional	501 (10.5)	59 (9.4)	24 (12.2)	66 (11.6)	337 (10.6)	
Metastatic	1603 (33.6)	192 (30.5)	64 (32.7)	181 (31.9)	1084 (34.2)	
Cancer-free but receiving adjuvant therapy	270 (5.7)	31 (4.9)	13 (6.6)	30 (5.3)	186 (5.9)	
Current cancer status^f^						
Stable	1466 (30.7)	215 (34.1)	47 (24.0)	166 (29.2)	977 (30.8)	.34
Progressing	601 (12.6)	85 (13.5)	26 (13.3)	71 (12.5)	388 (12.2)	
Responding to treatment	303 (6.4)	41 (6.5)	10 (5.1)	37 (6.5)	210 (6.6)	
Unknown	629 (13.2)	93 (14.8)	31 (15.8)	86 (15.1)	375 (11.8)	
Last known ECOG performance status score^g^						
0	1555 (32.6)	213 (33.8)	57 (29.1)	147 (25.9)	1066 (33.6)	<.001
1	1497 (31.4)	173 (27.5)	63 (32.1)	188 (33.1)	1020 (32.1)	
≥2	637 (13.4)	57 (9.0)	29 (14.8)	92 (16.2)	433 (13.6)	
Unknown	1077 (22.6)	186 (29.5)	47 (24.0)	141 (24.8)	653 (20.6)	
**Area-level SDOH**						
Census region of treatment oncology practice						
West	413 (8.7)	90 (14.3)	49 (25.0)	19 (3.3)	238 (7.5)	<.001
Midwest	1437 (30.1)	64 (10.2)	55 (28.1)	92 (16.2)	1177 (37.1)	
Northeast	620 (13.0)	66 (10.5)	21 (10.7)	100 (17.6)	416 (13.1)	
South	2293 (48.1)	410 (65.1)	71 (36.2)	357 (62.9)	1339 (42.2)	
Population density of treatment oncology practice						
Urban	4507 (94.5)	618 (98.1)	193 (98.5)	548 (96.5)	2963 (93.4)	<.001
Rural city or town	256 (5.4)	12 (1.9)	3 (1.5)	20 (3.5)	207 (6.5)	
Census region of patient’s primary residence^h^						
West	422 (8.9)	90 (14.3)	49 (25.0)	19 (3.3)	246 (7.8)	<.001
Midwest	1422 (29.8)	64 (10.2)	56 (28.6)	91 (16.0)	1163 (36.7)	
Northeast	617 (12.9)	66 (10.5)	21 (10.7)	100 (17.6)	412 (13.0)	
South	2305 (48.3)	410 (65.1)	70 (35.7)	357 (62.9)	1351 (42.6)	
Population density of patient’s primary residence^h^						
Urban	4091 (85.8)	585 (92.9)	177 (90.3)	523 (92.1)	2627 (82.8)	<.001
Rural city or town	675 (14.2)	45 (7.1)	19 (9.7)	44 (7.7)	545 (17.2)	
Median household income^h^^,^^i^						
<$43 125	821 (17.2)	218 (34.6)	29 (14.8)	214 (37.7)	336 (10.6)	<.001
$43 125-$54 047	1046 (21.9)	139 (22.1)	35 (17.9)	127 (22.4)	708 (22.3)	
$54 048-$68 446	1177 (24.7)	120 (19.0)	50 (25.5)	91 (16.0)	871 (27.5)	
≥$68 447	1403 (29.4)	136 (21.6)	72 (36.7)	120 (21.1)	986 (31.1)	
Population with only a high school diploma, %^h^^,^^i^						
<25.5	1501 (31.5)	196 (31.1)	94 (48.0)	186 (32.7)	930 (29.3)	<.001
25.5-33.7	1604 (33.6)	288 (45.7)	53 (27.0)	200 (35.2)	1011 (31.9)	
33.8-41.2	1067 (22.4)	118 (18.7)	33 (16.8)	147 (25.9)	726 (22.9)	
≥41.3	279 (5.9)	12 (1.9)	6 (3.1)	19 (3.3)	237 (7.5)	
Population aged ≤64 y with no health insurance, %^h^^,^^i^						
<4.8	639 (13.4)	40 (6.3)	28 (14.3)	30 (5.3)	507 (16.0)	<.001
4.8-8.8	1312 (27.5)	93 (14.8)	63 (32.1)	155 (27.3)	952 (30.0)	
8.9-14.7	1472 (30.9)	169 (26.8)	42 (21.4)	219 (38.6)	970 (30.6)	
≥14.8	1028 (21.6)	312 (49.5)	53 (27.0)	148 (26.1)	475 (15.0)	
Population reporting White race, %^h^^,^^i^						
≤77.3	1766 (37.0)	277 (44.0)	111 (56.6)	461 (81.2)	813 (25.6)	<.001
77.4-92.1	1845 (38.7)	303 (48.1)	60 (30.6)	83 (14.6)	1340 (42.2)	
92.2-97.4	683 (14.3)	33 (5.2)	14 (7.1)	6 (1.1)	600 (18.9)	
≥97.5	146 (3.1)	1 (0.2)	1 (0.5)	2 (0.4)	140 (4.4)	
Population reporting Hispanic ethnicity, %^h^^,^^i^						
<0.7	109 (2.3)	0	1 (0.5)	9 (1.6)	96 (3.0)	<.001
0.7-3.1	880 (18.5)	18 (2.9)	18 (9.2)	122 (21.5)	703 (22.2)	
3.2-9.5	1771 (37.1)	72 (11.4)	66 (33.7)	237 (41.7)	1327 (41.8)	
>9.5	1691 (35.5)	524 (83.2)	101 (51.5)	184 (32.4)	778 (24.5)	

Abbreviations: BMI, body mass index (calculated as weight in kilograms divided by height in meters squared); ECOG, Eastern Cooperative Oncology Group; SDOH, social determinants of health.

^a^ Missing data for the overall study sample were greater than 2% for BMI (200 [4.2%]), number of comorbidities requiring treatment within 12 months of COVID-19 diagnosis (99 [2.1%]), current cancer status (266 [8.1%]), median household income (321 [6.7%]), and percentage of population with only a high school diploma (317 [6.6%]), with no health insurance (317 [6.6%]), reporting White race (328 [6.9%]), and reporting Hispanic ethnicity (317 [6.6%]).

^b^ The racial and ethnic composition of the overall sample was 630 (13.2%) Hispanic, 177 (3.7%) non-Hispanic American Indian or Alaska Native, 196 (4.1%) non-Hispanic Asian American or Pacific Islander, 568 (11.9%) non-Hispanic Black, 3173 (66.5%) non-Hispanic White, and 24 (0.5%) other ethnicity (including 5 [0.1%] American Indian or Alaska Native, 8 [0.2%] Asian American or Pacific Islander, 5 [0.1%] Black, and 6 [0.1%] White patients).

^c^ The χ^2^ test was used for comparisons of proportions across race and ethnicity groups because there were no scenarios where more than 20% of expected cell counts were less than 5.

^d^ The following 2 sets of groups were combined: (1) less than 18.50 (underweight [n = 101]) and 18.50 to 25.00 (normal weight [n = 1215]) into less than 25.00 and (2) 35.00 to 39.99 (obesity class II [n = 517]) and at least 40.00 (obesity class III [n = 358]) into at least 35.00.

^e^ Gastrointestinal cancers include esophageal, stomach, liver, colorectal, and pancreatic cancers. Gynecologic cancers are cancers of the female and male reproductive organs. Genitourinary cancers include prostate, kidney, and bladder cancers. Lymphoid, hematopoietic, or related tissue cancers include lymphomas and leukemias. Respiratory or intrathoracic organ cancers include lung, bronchus, and heart cancers. Other cancers include those of the lip; oral cavity; pharynx; bone and articular cartilage; mesothelial and soft tissue; skin; eye, brain, and other parts of the central nervous system; thyroid and other endocrine glands; and unspecified cancers.

^f^ Patients without a solid tumor were ineligible to report cancer stage, and those without a solid tumor or cancer-free but receiving adjuvant therapy were ineligible to report current cancer status.

^g^ For ECOG performance status, 0 indicates fully active; 1, restricted in physically strenuous activity but ambulatory and able to carry out work of a light or sedentary nature; and 2 or greater, either capable of only limited self-care or completely disabled. Data were missing for 2 patients.

^h^ The SDOH variables were estimated based on census-level data for patient’s primary area of residence at cancer diagnosis.

^i^ To prevent reidentification of patients by way of their residential area, the SDOH variables were segmented into quartiles as shown.

### Outcomes

The primary outcome was delayed (≥14 days) or discontinued cancer treatment compared with timely receipt of cancer treatment (on schedule or within 14 days). Three cancer treatment modalities were examined: (1) surgery, (2) drug-based therapy (or pharmacotherapy), and (3) radiotherapy. A composite variable, any cancer treatment, was created to measure delay (≥14 days) or discontinuation of at least 1 treatment modality. We did not examine delayed or discontinued transplant or cellular therapies because too few patients were receiving or scheduled to receive these treatments (27 [0.6%]). The secondary outcome was time (in days) to restart pharmacotherapy, specifically for the first or only anticancer drug patients were receiving or scheduled to receive when they had positive test results for SARS-CoV-2. Almost 70% of patients were receiving or scheduled to receive pharmacotherapy, with 2391 (50.1%) receiving only 1 drug, 579 (12.1%) receiving 2, 181 (3.8%) receiving 3, and 59 (1.2%) receiving 4 or more, including chemotherapy (paclitaxel, carboplatin), targeted therapy (rituximab, ibrutinib), immunotherapy (pembrolizumab), and hormone therapy (letrozole, anastrozole, tamoxifen citrate).

### Exposures

The individual-level exposures (based on medical records) were sociodemographic factors, including race and ethnicity (Hispanic [inclusive of any race], non-Hispanic American Indian or Alaska Native, non-Hispanic Asian American or Pacific Islander, non-Hispanic Black, non-Hispanic White, or Other), sex (men or women), and age at confirmed positive SARS-CoV-2 test result (<50, 50-59, 60-69, or ≥70 years). Other individual-level covariates were clinical factors, including body mass index (BMI; calculated as weight in kilograms divided by height in meters squared), number of comorbidities requiring active treatment within 12 months of confirmed positive SARS-CoV-2 test result (0, 1, 2, or ≥3), cancer type (breast, gastrointestinal, gynecologic and/or genitourinary, lymphoid, hematopoietic, related tissue, respiratory and/or intrathoracic organ, or other), solid tumor status (no or yes), cancer stage for solid tumor cancers (local, regional, metastatic, or cancer-free but receiving adjuvant therapy), and last known performance status score (0, 1, ≥2, or unknown based on the Eastern Cooperative Oncology Group [ECOG] scale). Information on tobacco use (current, former, never, or unsure) and current cancer status (stable, progressing, responding to treatment, or unknown) was also collected, but neither variable was included in multivariable models since the former was not associated with any treatment outcome and the latter was highly correlated with cancer stage.

The area-level SDOH (from the Agency for Healthcare Research and Quality’s Social Determinants of Health Database, specifically zip code–level data derived from the Census Bureau’s American Community Survey 5-year estimates) were SDOH in the patients’ residential area, including census region (West, Midwest, Northeast, or South), population density (urban or rural city or town), median household income, and proportion of population with only a high school diploma, 64 years or younger without health insurance, reporting White race, and reporting Hispanic ethnicity. To prevent patient reidentification, the SDOH variables were segmented into quartiles. The ASCO COVID-19 Registry collected data on treatment oncology practices’ census region and population density, but these variables were highly correlated with those at the patient’s primary residence and thus excluded from the analysis. Further, multivariable models only included the proportion of the population reporting White race, not Hispanic ethnicity, because the two are correlated.

### Statistical Analysis

Patient and cancer treatment characteristics were compared across race and ethnicity using χ^2^ or Fisher exact tests and analysis of variance to identify significant differences for categorical and continuous variables, respectively. Independent relative risk (RR) regression models^20,21,22,23^ were used to assess factors associated with delayed or discontinued receipt of surgery, pharmacotherapy, radiotherapy, and any cancer treatment using the generalized linear model procedure with a Poisson distribution, log link, and robust error variances, separately for individual-level and area-level SDOH. Relative risks for models with individual-level factors were tabulated, while RRs for models with area-level SDOH were graphed. Regression analysis for RRs with individual-level factors used a Bonferroni-adjusted 2-sided *P* value of .004 (α = .05/12) because we tested 12 comparisons among race and ethnicity, sex, and age with 4 treatment outcomes (any cancer treatment, surgery, pharmacotherapy, and radiotherapy). For the area-level factors, we used a Bonferroni-adjusted *P* value of .002 (α = .05/24) since we tested 24 comparisons for the 6 area-level SDOH factors with the 4 cancer treatment outcomes.

Cox proportional hazards regression analysis was used to investigate the association of race and ethnicity with time to restart of pharmacotherapy following a confirmed positive test result for SARS-CoV-2, adjusting for age and cancer type. Sex, BMI, tobacco use, number of comorbidities, cancer stage, and ECOG performance status score were not associated with this outcome and therefore were not included in the Cox proportional hazards regression model as confounders. We used SPSS, version 26.0 (IBM Corp) to compute descriptive statistics; Stata, version 16.1 (StataCorp LLC) for regression analyses; and Prism, version 9.3.1 (GraphPad) for RR visualization of area-level SDOH.

## Results

### Patient Characteristics

A total of 4768 patients with cancer (2756 women [57.8%] and 2012 men [42.2%]; 1558 aged ≥70 years [32.7%]) were included in the analysis (630 [13.2%] Hispanic, 177 [3.7%] non-Hispanic American Indian or Alaska Native, 196 [4.1%] non-Hispanic Asian American or Pacific Islander, 568 [11.9%] non-Hispanic Black, 3173 [66.5%] non-Hispanic White, and 24 [0.5%] Other). Patients had a laboratory-confirmed SARS-CoV-2 infection, and positive SARS-CoV-2 test results were more frequent among patients 50 years or older (3910 [82.0%]) (Table 1). Racial and ethnic differences were observed for some sociodemographic and health characteristics: larger proportions of members of racial and ethnic minority groups were younger (<50 years: 202 [32.1%] Hispanic, 47 [24.0%] non-Hispanic Asian American or Pacific Islander, and 138 [24.3%] non-Hispanic Black vs. 434 [13.7%] non-Hispanic White) and residents of urban (585 [92.9%] Hispanic, 177 [90.3%] non-Hispanic Asian American or Pacific Islander, and 523 [92.1%] non-Hispanic Black vs. 2627 [82.8%] non-Hispanic White) and socioeconomically disadvantaged areas (lowest quartile of median household income at patient’s primary area of residence, <$43 125: 218 [34.6%] Hispanic, 29 [14.8%] non-Hispanic Asian American or Pacific Islander, and 214 [37.7%] non-Hispanic Black vs 336 [10.6%] non-Hispanic White; highest quartile of percentage of population with no health insurance, ≥14.8%: 312 [49.5%] Hispanic, 53 [27.0%] non-Hispanic Asian American or Pacific Islander, and 148 [26.1%] non-Hispanic Black vs 475 [15.0%] non-Hispanic White) (*P* < .001 for all). Larger proportions of non-Hispanic Black patients had BMIs of 35.00 or greater (133 [23.4%]; *P* = .009), were current smokers (66 [11.6%]; *P* < .001), and had poorer ECOG performance status scores (≥2, 92 [16.2%]; *P* < .001) than all other racial and ethnic groups.

Deceased patients (n = 453) were excluded from the analytic sample and consisted of a higher proportion of members of racial and ethnic minority groups (51 [11.3%] Hispanic, 21 [4.6%] non-Hispanic American Indian or Alaska Native, 26 [5.7%] non-Hispanic Asian American or Pacific Islander, 80 [17.7%] non-Hispanic Black, 273 [60.3%] non-Hispanic White, and 2 [0.4%] other race or ethnicity) when compared with those included in the study sample (*P* = .003) (eTable in Supplement 1). In contrast to living patients, a larger proportion of the deceased were male (234 [51.7%] vs 2012 [42.2%]), 70 years or older (248 [54.7%] vs 1558 [32.7%]), and Northeast residents (136 [30.0%] vs 620 [13.0%]) and had resided in areas with fewer uninsured (eg, <4.8%, 77 [17.0%] vs 639 [13.4%]) and White (eg, ≤77.3%, 221 [48.8%] vs 1766 [37.0%]) residents (*P* ≤ .001).

More non-Hispanic White patients (2289 [72.1%]) were receiving or scheduled to receive pharmacotherapy compared with members of racial and ethnic minority groups (eg, Hispanic, 375 [59.5%]; *P* < .001) (Table 2). Relative to other groups, non-Hispanic Black patients more frequently experienced pharmacotherapy delays (183 [32.2%]), while non-Hispanic White patients more frequently received timely pharmacotherapy (1386 [43.7%]) and were less likely to have pharmacotherapy discontinued (91 [2.9%]) (*P* < .001). Fewer Hispanic (175 [27.8%]), non-Hispanic Asian American or Pacific Islander (50 [25.5%]), and non-Hispanic Black patients (160 [28.2%]) than non-Hispanic White patients (1016 [32.0%]) were scheduled to receive at least 1 dose of pharmacotherapy (*P* = .009). Differences were not observed for timely receipt of surgery or radiotherapy post SARS-CoV-2 infection. COVID-19 was the key reason for treatment delay or discontinuation, while lack of clinical resources was not a major factor (eFigure 1 in Supplement 1).

**Table 2.** Characteristics of Cancer Treatment Following Confirmed SARS-Cov-2 Test Result

Cancer treatment characteristic	Patient group^a^					*P* value^b^
	Overall (N = 4768)	Race and ethnicity				
		Hispanic (n = 630)	Non-Hispanic Asian American or Pacific Islander (n = 196)	Non-Hispanic Black (n = 568)	Non-Hispanic White (n = 3173)	
Patient enrolled in therapeutic cancer clinical trial						
No	4372 (91.7)	595 (94.4)	166 (84.7)	504 (88.7)	2926 (92.2)	.22
Yes	140 (2.9)	18 (2.9)	8 (4.1)	22 (3.9)	84 (2.6)	
Patient enrolled in hospice						
No	4552 (95.5)	611 (97.0)	178 (90.8)	526 (92.6)	3044 (95.9)	.72
Yes	38 (0.8)	6 (1.0)	1 (0.5)	6 (1.1)	23 (0.7)	
**Surgery**						
Patient had surgery to remove cancer in last 6 wk						
No	4420 (92.7)	592 (94.0)	173 (88.3)	514 (90.5)	2955 (93.1)	.98
Yes	128 (2.7)	18 (2.9)	4 (2.0)	15 (2.6)	84 (2.6)	
Patient receiving or scheduled to receive surgery in 0-6 wk						
No	4560 (95.6)	600 (95.2)	184 (93.9)	539 (94.9)	3040 (95.8)	.48
Yes	208 (4.4)	30 (4.8)	12 (6.1)	29 (5.1)	133 (4.2)	
Receipt on schedule or within 14 d	74 (1.6)	13 (2.1)	5 (2.6)	6 (1.1)	48 (1.5)	.38
Receipt discontinued or canceled	3 (0.1)	1 (0.2)	0	0	2 (0.1)	
Receipt delayed ≥14 d	131 (2.7)	16 (2.5)	7 (3.6)	23 (4.0)	83 (2.6)	
No. of days surgery delayed, mean (SD)	26.1 (46.9)	20.5 (31.2)	22.4 (21.8)	53.6 (94.1)	20.5 (27.9)	.08
**Pharmacotherapy**						
Patient receiving or scheduled to receive pharmacotherapy						
No	1463 (30.7)	255 (40.5)	69 (35.2)	181 (31.9)	884 (27.9)	<.001
Yes	3305 (69.3)	375 (59.5)	127 (64.8)	387 (68.1)	2289 (72.1)	
Receipt on schedule or within 14 d	1914 (40.1)	201 (31.9)	79 (40.3)	173 (30.5)	1386 (43.7)	<.001
Receipt discontinued or canceled	154 (3.2)	19 (3.0)	11 (5.6)	31 (5.5)	91 (2.9)	
Receipt delayed ≥14 d	1236 (25.9)	155 (24.6)	36 (18.4)	183 (32.2)	812 (25.6)	
No. of drug-based agents received or to receive						
1	2391 (50.1)	295 (46.8)	86 (43.9)	255 (44.9)	1659 (52.3)	.06
2	579 (12.1)	61 (9.7)	20 (10.2)	76 (13.4)	398 (12.5)	
3	181 (3.8)	10 (1.6)	11 (5.6)	28 (4.9)	127 (4.0)	
≥4	59 (1.2)	5 (0.8)	3 (1.5)	6 (1.1)	44 (1.4)	
First anticancer drug therapy						
Resumed after confirmed SARS-CoV-2 test result	1812 (38.0)	230 (36.5)	62 (31.6)	212 (37.3)	1223 (38.5)	.05
No. of days to restart, mean (SD)	46.8 (51.8)	45.5 (34.8)	56.8 (88.7)	51.8 (50.3)	46.8 (54.1)	.62
Scheduled receipt after last recorded dose						
No, patient completed regimen	185 (3.9)	36 (5.7)	9 (4.6)	29 (5.1)	105 (3.3)	.009
No, patient stopped prior to completing regimen	119 (2.5)	13 (2.1)	3 (1.5)	18 (3.2)	81 (2.6)	
Yes, patient will receive ≥1 dose	1474 (30.9)	175 (27.8)	50 (25.5)	160 (28.2)	1016 (32.0)	
Second anticancer drug therapy						
Resumed after confirmed SARS-CoV-2 test result	421 (8.8)	42 (6.7)	14 (7.1)	63 (11.1)	281 (8.9)	.25
No. of days to restart, mean (SD)	49.4 (52.3)	43.9 (25.1)	49.0 (51.8)	50.6 (50.6)	51.6 (57.8)	.92
Scheduled receipt after last recorded dose						
No, patient completed regimen	46 (1.0)	7 (1.1)	3 (1.5)	9 (1.6)	26 (0.8)	.38
No, patient stopped prior to completing regimen	25 (0.5)	1 (0.2)	1 (0.5)	3 (0.5)	19 (0.6)	
Yes, patient will receive ≥1 dose	337 (7.1)	33 (5.2)	10 (5.1)	48 (8.5)	228 (7.2)	
**Radiotherapy**						
Patient receiving or scheduled to receive radiotherapy						
No	4427 (92.8)	596 (94.6)	175 (89.3)	520 (91.5)	2950 (93.0)	.04
Yes	341 (7.2)	34 (5.4)	21 (10.7)	48 (8.5)	223 (7.0)	
Receipt on schedule or within 14 d	202 (4.2)	21 (3.3)	11 (5.6)	28 (4.9)	131 (4.1)	.86
Receipt discontinued or canceled	23 (0.5)	1 (0.2)	3 (1.5)	3 (0.5)	15 (0.5)	
Receipt delayed at least 14 d	116 (2.4)	12 (1.9)	7 (3.6)	17 (3.0)	77 (2.4)	
Resumed after COVID-19 diagnosis	175 (3.7)	15 (2.4)	12 (6.1)	25 (4.4)	117 (3.7)	.26
Time to restart, mean (SD), d	22.5 (24.6)	40.6 (49.8)	28.0 (26.8)	26.3 (19.0)	16.7 (17.0)	.13
Scheduled receipt after last recorded dose						
No, patient completed regimen	111 (2.3)	8 (1.3)	7 (3.6)	16 (2.8)	76 (2.4)	.87
No, patient stopped prior to completing regimen	0	0	0	0	0	
Yes, patient will receive ≥1 dose	50 (1.0)	4 (0.6)	4 (2.0)	7 (1.2)	34 (1.1)	
Unknown status of schedule	12 (0.3)	2 (0.3)	0	2 (0.4)	7 (0.2)	

^a^ The racial and ethnic composition of the overall sample was 630 (13.2%) Hispanic, 177 (3.7%) non-Hispanic American Indian or Alaska Native, 196 (4.1%) non-Hispanic Asian American or Pacific Islander, 568 (11.9%) non-Hispanic Black, 3173 (66.5%) non-Hispanic White, and 24 (0.5%) other race or ethnicity (including 5 [0.1%] American Indian or Alaska Native, 8 [0.2%] Asian American or Pacific Islander, 5 [0.1%] Black, and 6 [0.1%] White patients). Unless otherwise indicated, data are expressed as No. (%) of patients.

^b^ The χ^2^ test was used for comparisons of proportions where 20% or less of expected cell counts were less than 5, and the Fisher exact test was used for comparisons of proportions where more than 20% of expected cell counts were less than 5. Analysis of variance was used to compare means of continuous variables across race and ethnicity categories.

### Cancer Treatment Following a Confirmed Positive SARS-CoV-2 Test Result

Non-Hispanic Black patients were more likely to experience delays or discontinuations of at least 14 days for any cancer treatment (RR, 1.35 [95% CI, 1.22-1.49]; *P* < .001) and pharmacotherapy (RR, 1.37 [95% CI, 1.23-1.52]; *P* < .001) relative to non-Hispanic White patients (Table 3). Although not significant with multiple comparisons correction (adjusted *P* > .004), Hispanic patients had a higher likelihood of delay or discontinuation of any cancer treatment (RR, 1.14 [95% CI, 1.00-1.28]; *P* = .04) and pharmacotherapy (RR, 1.17 [95% CI, 1.03-1.33]; *P* = .02) compared with non-Hispanic White patients. Race and ethnicity were not associated with surgery or radiotherapy delays or discontinuation. Sex and age were not associated with delay or discontinuation of any treatment modality (RR for 60-69 vs <50 years, 1.69 [95% CI, 1.03-2.78; *P* = .04]; RR for ≥70 vs <50 years, 1.64 [95% CI, 0.99-2.73; *P* = .06]).

**Table 3.** Multivariable-Adjusted RR Regression Analysis of Individual-Level Factors Associated With Delayed or Discontinued Cancer Treatment Following Confirmed SARS-Cov-2 Test Result

Factor	Receipt of cancer treatment delayed or discontinued							
	Any cancer treatment (N = 3323)		Surgery (n = 190)		Pharmacotherapy (n = 3030)		Radiotherapy (n = 315)	
	RR (95% CI)	*P* value^a^	RR (95% CI)	*P* value^a^	RR (95% CI)	*P* value^a^	RR (95% CI)	*P* value^a^
**Sociodemographic**								
Race and ethnicity								
Hispanic	1.14 (1.00-1.28)	.04	0.89 (0.59-1.33)	.56	1.17 (1.03-1.33)	.02	1.11 (0.68-1.83)	.67
Non-Hispanic Asian American or Pacific Islander	0.91 (0.73-1.13)	.38	0.79 (0.44-1.41)	.43	0.91 (0.71-1.16)	.45	1.29 (0.80-2.09)	.30
Non-Hispanic Black	1.35 (1.22-1.49)	<.001	1.17 (0.91-1.52)	.23	1.37 (1.23-1.52)	<.001	1.06 (0.70-1.61)	.78
Non-Hispanic White	1 [Reference]	NA	1 [Reference]	NA	1 [Reference]	NA	1 [Reference]	NA
Sex								
Men	0.99 (0.90-1.07)	.74	0.99 (0.76-1.31)	.97	0.98 (0.89-1.07)	.60	1.01 (0.73-1.40)	.94
Women	1 [Reference]	NA	1 [Reference]	NA	1 [Reference]	NA	1 [Reference]	NA
Age, y								
<50	1 [Reference]	NA	1 [Reference]	NA	1 [Reference]	NA	1 [Reference]	NA
50-59	1.14 (1.00-1.30)	.05	1.20 (0.86-1.67)	.28	1.10 (0.96-1.27)	.18	1.41 (0.83-2.41)	.20
60-69	1.04 (0.91-1.19)	.55	1.29 (0.90-1.87)	.17	0.99 (0.86-1.14)	.92	1.69 (1.03-2.78)	.04
≥70	1.04 (0.91-1.18)	.61	1.34 (0.95-1.90)	.10	1.00 (0.87-1.15)	>.99	1.64 (0.99-2.73)	.06
Health history								
BMI	1.00 (0.99-1.00)	.32	1.01 (0.99-1.02)	.41	0.99 (0.99-1.00)	.05	1.01 (0.99-1.03)	.18
No. of comorbidities								
0	1 [Reference]	NA	1 [Reference]	NA	1 [Reference]	NA	1 [Reference]	NA
1	1.04 (0.94-1.15)	.45	0.91 (0.67-1.24)	.57	1.09 (0.98-1.22)	.11	0.86 (0.58-1.25)	.42
2	1.11 (0.99-1.24)	.08	0.96 (0.71-1.30)	.80	1.15 (1.02-1.30)	.02	1.06 (0.73-1.54)	.77
≥3	1.19 (1.05-1.35)	.008	0.85 (0.60-1.19)	.34	1.23 (1.07-1.42)	.003	1.00 (0.67-1.49)	.98
**Cancer status**								
Cancer type								
Breast	1 [Reference]	NA	1 [Reference]	NA	1 [Reference]	NA	1 [Reference]	NA
Gastrointestinal	1.29 (1.14-1.47)	<.001	1.06 (0.78-1.45)	.69	1.34 (1.17-1.54)	<.001	1.04 (0.68-1.59)	.85
Gynecologic or genitourinary	1.02 (0.88-1.18)	.81	0.91 (0.67-1.23)	.52	1.02 (0.87-1.21)	.78	0.66 (0.40-1.07)	.09
Lymphoid, hematopoietic, or related tissue	1.42 (1.17-1.71)	<.001	0.67 (0.24-1.87)	.45	1.54 (1.26-1.87)	<.001	0.42 (0.07-2.59)	.35
Respiratory or intrathoracic organ	1.10 (0.95-1.28)	.19	0.90 (0.46-1.73)	.74	1.18 (1.01-1.39)	.04	0.84 (0.54-1.31)	.44
Other	0.95 (0.81-1.12)	.58	0.74 (0.49-1.12)	.16	1.00 (0.83-1.20)	.97	0.71 (0.46-1.09)	.11
Cancer stage								
Local	1 [Reference]	NA	1 [Reference]	NA	1 [Reference]	NA	1 [Reference]	NA
Regional	1.24 (1.08-1.42)	.002	1.12 (0.87-1.46)	.37	1.36 (1.15-1.60)	<.001	1.05 (0.74-1.49)	.78
Metastatic	1.17 (1.05-1.30)	.005	0.88 (0.62-1.25)	.47	1.35 (1.19-1.55)	<.001	1.11 (0.78-1.56)	.56
Cancer-free but receiving adjuvant therapy	0.55 (0.41-0.73)	<.001	0.71 (0.18-2.78)	.63	0.56 (0.40-0.79)	.001	0.78 (0.42-1.47)	.45
No solid tumor	0.79 (0.66-0.96)	.02	0.81 (0.36-1.80)	.60	0.89 (0.72-1.09)	.26	0.69 (0.18-2.59)	.58
Last known ECOG performance status score^b^								
0	1 [Reference]	NA	1 [Reference]	NA	1 [Reference]	NA	1 [Reference]	NA
1	1.10 (1.00-1.22)	.06	1.29 (0.99-1.68)	.06	1.09 (0.98-1.22)	.10	0.92 (0.63-1.33)	.66
≥2	1.24 (1.10-1.40)	.001	1.13 (0.65-1.95)	.67	1.24 (1.09-1.41)	.001	0.99 (0.65-1.51)	.97
Unknown	1.15 (1.02-1.29)	.02	1.23 (0.94-1.62)	.13	1.12 (0.99-1.27)	.08	1.09 (0.77-1.55)	.62

Abbreviations: BMI, body mass index; ECOG, Eastern Cooperative Oncology Group; NA, not applicable; RR, risk ratio.

^a^ We conducted RR regression using a Poisson distribution, log link, and robust error variances to obtain RRs that examined the association among sociodemographic, health history, and cancer status factors with receipt of delayed or discontinued cancer treatment (as opposed to on schedule or timely cancer treatment), following a confirmed SARS-Cov-2 test result.

^b^ For ECOG performance status, 0 indicates fully active; 1, restricted in physically strenuous activity but ambulatory and able to carry out work of a light or sedentary nature; and 2 or greater, either capable of only limited self-care or completely disabled.

Generally, area-level SDOH were not associated with delayed or discontinued surgery, pharmacotherapy, and radiotherapy after Bonferroni correction (adjusted *P* > .002) (eFigure 2 in Supplement 1). For any treatment, residents of the Midwest vs South were 18% less likely to experience delays or discontinuations (RR, 0.82 [95% CI, 0.74-0.91]; *P* < .001), and residents of areas with larger White populations (RR for 77.4%-92.1% vs ≤77.3%, 0.87 [95% CI, 0.79-0.95]; *P* = .002) were 13% less likely to experience delays or discontinuations. Results were not significant for residents of areas where less than 25.5% vs 41.3% or more of the population had only a high school diploma (RR, 1.26 [95% CI, 1.01-1.57]; *P* = .04), lower median household incomes (RR for <$43 125 vs ≥$68 447, 1.20 [95% CI, 1.02-1.42]; *P* = .03), and fewer uninsured residents (RR for <4.8% vs ≥14.8%, 1.25 [95% CI, 1.05-1.49; *P* = .01]; RR for 4.8%-8.8% vs ≥14.8%, 1.24 [95% CI, 1.08-1.42; *P* = .002]) (adjusted *P* > .002).

### Restart of Cancer Treatment Following a Confirmed Positive SARS-CoV-2 Test Result

The time to restart pharmacotherapy was 19% longer (HR, 0.81 [95% CI, 0.67-0.97]; *P* = .03) for non-Hispanic Black than non-Hispanic White patients (Figure). Results were not statistically significant for non-Hispanic Asian American or Pacific Islander (hazard ratio [HR], 0.79 [95% CI, 0.46-1.35]; *P* = .39) and Hispanic patients (HR, 0.87 [95% CI, 0.71-1.05]; *P* = .15). In sex-stratified analysis (eFigure 3 in Supplement 1), no significant differences were observed. However, for men, pharmacotherapy restart rate appeared to be 40% longer for non-Hispanic Asian American or Pacific Islander patients (HR, 0.60 [95% CI, 0.27-1.33]; *P* = .21), 22% longer for Hispanic patients (HR, 0.78 [95% CI, 0.58-1.06]; *P* = .11), and 24% longer for non-Hispanic Black patients (HR, 0.76 [95% CI, 0.58-1.01]; *P* = .06) relative to non-Hispanic White patients while adjusting for age and cancer type (eFigures 4 and 5 in Supplement 1).

**Figure.**
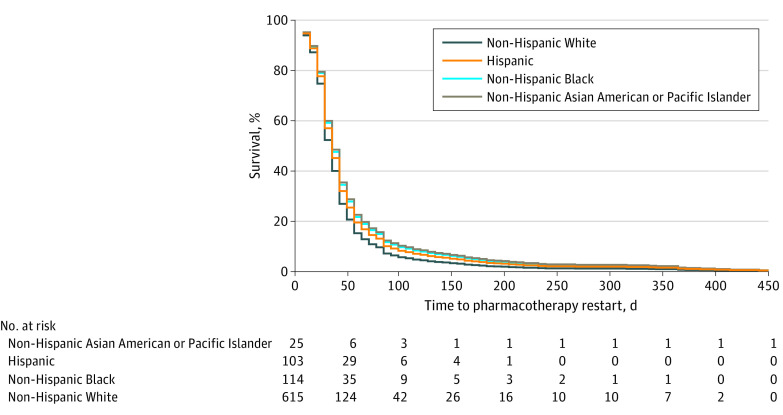
Adjusted Survival Curves of Time to Restart Pharmacotherapy Following a Confirmed Positive SARS-CoV-2 Test Result, Stratified by Race and Ethnicity Includes 857 patients with cancer. A Cox proportional hazards regression analysis was performed to assess differences in the restart rate of the first or only anticancer drug treatment patients were scheduled to receive, by race and ethnicity, with adjustment for age and cancer type. Compared with non-Hispanic White patients, the restart rate was 19% less (hazard ratio [HR], 0.81 [95% CI, 0.67-0.97]; *P* = .03) for non-Hispanic Black patients, 13% less (HR, 0.87 [95% CI, 0.71-1.05]; *P* = .15) for Hispanic patients, and 21% less (HR, 0.79 [95% CI, 0.46-1.35]; *P* = .39) for non-Hispanic Asian American or Pacific Islander patients.

## Discussion

We examined associations between individual-level and area-level SDOH factors and cancer treatment delay or discontinuation post SARS-CoV-2 infection in a racially and ethnically diverse sample during the COVID-19 pandemic. Pandemic-related cancer treatment delays have the potential to increase cancer morbidity and mortality rates and widen cancer survival inequalities for years to come.^15,24,25,26,27,28,29,30^ Our findings highlight that relative to non-Hispanic White patients, non-Hispanic Black patients were 35% and 37% more likely to experience delay or discontinuation of any cancer treatment and pharmacotherapy, respectively. Compared with non-Hispanic White patients, Hispanic patients were 14% and 17% more likely to experience delay or discontinuation of any cancer treatment and pharmacotherapy, respectively. These findings corroborate previous reports of delays in the time-to-treatment continuum among patients with cancer from racial and ethnic minority groups.^13,31,32,33,34,35,36^ Treatment patterns during the COVID-19 pandemic support higher rates of noninitiation, discontinuation, and nonadherence to adjuvant endocrine therapy among patients with cancer from racial and ethnic minority groups.^13,15,31,32,34,35,36^ Evidence shows that non-Hispanic Black and Hispanic individuals with cancer more frequently experience treatment delays and less frequently receive definitive treatment than non-Hispanic White individuals^31,37,38,39^; these racial and ethnic minority groups, older patients, those with lower SES, and those covered by Medicaid also have used telemedicine for cancer care less frequently during the pandemic.^31,32,40,41,42^

Our study findings further suggest that place—geography and area-level SDOH—might be associated with who is more likely to experience treatment delay or discontinuation, which may relate to geographic and transportation issues, resource constraints, etc.^43,44^ This is consistent with the understanding that residents of socioeconomically disadvantaged areas are more likely to experience various forms of health inequalities, independent of individual-level factors associated with those outcomes.^44,45,46^ For example, lower rates of survival of prostate cancer, higher rates of infant mortality, and higher rates of premature death are associated with living in areas with predominantly racial and ethnic minority groups.^47,48,49^ With COVID-19, these spatially linked metrics are important given that the behaviors and circumstances of community members influence the spread of infection.^50^

COVID-19 pandemic inequities experienced by some patients with cancer in our study and more broadly may be explained by a number of factors: (1) metropolitan areas were the epicenters of the COVID-19 pandemic, and in the US, racial and ethnic minority groups are more concentrated in urban areas; (2) lower-income apartment complexes, which are ill suited for optimal physical distancing procedures, house a disproportionately high concentration of these groups; (c) racial and ethnic minority groups are overrepresented in essential jobs excluded from shelter-in-place rules, such as in health care, building and maintenance, delivery services, and public transportation; (d) workers in essential services who are members of racial and ethnic minority groups rely more on public transportation, making physical distancing more difficult and increasing COVID-19 exposures; and (e) racial and ethnic minority groups more often live in multigenerational households, exposing vulnerable seniors to greater risks.^14,51^ Also, due to the COVID-19 pandemic’s economic impact, racial and ethnic minority groups were frequently represented among newly unemployed and uninsured or underinsured groups.^14,52,53^ This is important because almost 40% of patients with cancer reported that COVID-19 significantly impacted their financial status, impairing their capacity to pay for health care^54^ and increasing the likelihood of delayed care.

Understanding the broad-reaching effects of the COVID-19 pandemic and the impact of SDOH are critical for identifying opportunities to improve clinical practice and develop cancer care delivery models to improve outcomes in vulnerable groups. Our results suggest that previously existing cancer inequities may be exacerbated by suboptimal cancer care, specifically delayed pharmacotherapy, among non-Hispanic Black and Hispanic patients with cancer following the COVID-19 pandemic.^15^ These findings coincide with data from the COVID-19 and Cancer Outcomes Study,^17^ demonstrating more than 53% higher odds of delayed cancer care among patients who are members of racial and ethnic minority groups vs White patients during the pandemic.

### Limitations

Several limitations should be considered in the interpretation of our findings. First, power was limited to detect associations with surgery, radiotherapy, and multidrug pharmacotherapy (in the analysis examining restart of pharmacotherapy), as few patients were receiving or scheduled to receive these treatments at the time of their positive SARS-CoV-2 test result. Exclusion of deceased patients may have also impacted power, particularly among non-Hispanic Asian American or Pacific Islander and Hispanic patients, and contributed to selection bias given the differences observed between deceased patients and those in the analytical sample (eg, age, sex, area-level SDOH). Our use of a registry-based sample may have also contributed to selection bias because some patients with cancer may not have sought out a laboratory SARS-CoV-2 test, especially if they were experiencing COVID-19 symptoms, while others may have had false-negative test results, making them ineligible to participate in this study. Moreover, patients with cancer in the registry are already connected to oncology care and have survived long enough to receive treatment and therefore may not be representative of all patients with cancer in the general population. Other potential limitations include passive data collection (rather than self-report [ie, for race and ethnicity]), which increased the likelihood of data missingness, including data on potential confounders of the associations of interest, particularly the associations with area-level SDOH.

## Conclusions

The COVID-19 pandemic has significantly disrupted health care delivery and laid bare structural inequities, further motivating a period of racial reckoning and social justice for public health in the US. The findings of this cohort study suggest that SDOH are associated with racial and ethnic health care inequities among patients with cancer who become infected with SARS-CoV-2. These SDOH include structural racism and discrimination that result in individual-level and neighborhood-level deprivation from the long-term impacts of civil rights laws, legal racial discrimination, economic instability, police violence and overpolicing, and/or residential segregation and housing discrimination experienced by racial and ethnic minority groups.^55,56^

The consequences of cancer treatment decisions made throughout the COVID-19 pandemic, but particularly early on, cannot yet be fully examined but will likely include an exacerbation of cancer survival inequities by race and ethnicity and other SDOH. This study’s findings on at-risk patients can be used by oncology clinicians and public health professionals to inform plans to improve care across the cancer continuum and among patients with cancer undergoing active treatment. It is our hope that these data contribute to the development and implementation of multilevel interventions targeting microlevel and macrolevel determinants to reduce the likelihood of delayed oncology care among vulnerable patient populations during public health emergencies.

## Notes

Supplement 1. **eTable.** Sociodemographic and SDOH Characteristics of Patients With Cancer in the ASCO Registry at Entry into the Cohort (Date of Confirmed SARS-CoV-2 Test), by Vital Status (n = 5221) **eFigure 1.** Primary Reason for Delay or Discontinuation of Cancer Treatment Following a Confirmed Positive SARS-CoV-2 Test Result (n = 4768) **eFigure 2.** Relative Risk Regression Using a Poisson Distribution, Log Link, and Robust Error Variances **eFigure 3.** Adjusted Survival Curves of Time to Restart Pharmacotherapy Following a Confirmed Positive SARS-CoV-2 Test Result, Stratified by Race and Ethnicity, Separately for Men (n = 351) and Women (n = 506) **eFigure 4.** Adjusted Survival Curves of Time to Restart Pharmacotherapy Following a Confirmed Positive SARS-CoV-2 Test Result, Stratified by Age at COVID-19 Diagnosis (n = 857) **eFigure 5.** Adjusted Survival Curves of Time to Restart Pharmacotherapy Following a Confirmed Positive SARS-CoV-2 Test Result, Stratified by Cancer Type (n = 857)

Supplement 2.
Data Sharing Statement
