# Supplementary material for: Evaluation of Inequities in Cancer Treatment Delay or Discontinuation Following SARS-CoV-2 Infection
Source: JAMA Netw Open. 2023 Jan 13;6(1):e2251165. doi: 10.1001/jamanetworkopen.2022.51165 (PMC9856904; doi:10.1001/jamanetworkopen.2022.51165)
Supplement: Supplement 1. — eTable. Sociodemographic and SDOH Characteristics of Patients With Cancer in the ASCO Registry at Entry into the Cohort (Date of Confirmed SARS-CoV-2 Test), by Vital Status (n = 5221) eFigure 1. Primary Reason for Delay or Discontinuation of Cancer Treatment Following a Confirmed Positive SARS-CoV-2 Test Result (n = 4768) eFigure 2. Relative Risk Regression Using a Poisson Distribution, Log Link, and Robust Error Variances eFigure 3. Adjusted Survival Curves of Time to Restart Pharmacotherapy Following a Confirmed Positive SARS-CoV-2 Test Result, Stratified by Race and Ethnicity, Separately for Men (n = 351) and Women (n = 506) eFigure 4. Adjusted Survival Curves of Time to Restart Pharmacotherapy Following a Confirmed Positive SARS-CoV-2 Test Result, Stratified by Age at COVID-19 Diagnosis (n = 857) eFigure 5. Adjusted Survival Curves of Time to Restart Pharmacotherapy Following a Confirmed Positive SARS-CoV-2 Test Result, Stratified by Cancer Type (n = 857) [file jamanetwopen-e2251165-s001.pdf]

Supplementary Online Content

Llanos AAM, Ashrafi A, Ghosh N, et al. Evaluation of inequities in cancer treatment delay or discontinuation following SARS-CoV-2 infection. *JAMA Netw Open*. 2023;6(1):e2251165. doi:10.1001/jamanetworkopen.2022.51165

eTable. Sociodemographic and SDOH Characteristics of Patients With Cancer in the ASCO Registry at Entry into the Cohort (Date of Confirmed SARS-CoV-2 Test), by Vital Status (n = 5221)
eFigure 1. Primary Reason for Delay or Discontinuation of Cancer Treatment Following a Confirmed Positive SARS-CoV-2 Test Result (n = 4768)
eFigure 2. Relative Risk Regression Using a Poisson Distribution, Log Link, and Robust Error Variances
eFigure 3. Adjusted Survival Curves of Time to Restart Pharmacotherapy Following a Confirmed Positive SARS-CoV-2 Test Result, Stratified by Race and Ethnicity, Separately for Men (n = 351) and Women (n = 506)
eFigure 4. Adjusted Survival Curves of Time to Restart Pharmacotherapy Following a Confirmed Positive SARS-CoV-2 Test Result, Stratified by Age at COVID-19 Diagnosis (n = 857)
eFigure 5. Adjusted Survival Curves of Time to Restart Pharmacotherapy Following a Confirmed Positive SARS-CoV-2 Test Result, Stratified by Cancer Type (n = 857)

This supplementary material has been provided by the authors to give readers additional information about their work.

© 2023 Llanos AAM et al. *JAMA Network Open*.

eTable. Sociodemographic and SDOH Characteristics of Patients With Cancer in the ASCO Registry at Entry into the Cohort (Date of Confirmed SARS-CoV-2 Test), by Vital Status (n = 5221)

PATIENT CHARACTERISTICS[‡]	LIVING Included N (%)	DECEASED Excluded N (%)	*P* Value[†]
**TOTAL**	4768 (100.0)	453 (100.0)	
**PATIENT-LEVEL SOCIODEMOGRAPHICS**			
**Race and ethnicity**			.003
AAPI	196 (4.1)	26 (5.7)	
AIAN	177 (3.7)	21 (4.6)	
Hispanic	630 (13.2)	51 (11.3)	
NHB	568 (11.9)	80 (17.7)	
NHW	3173 (66.5)	273 (60.3)	
Other	24 (0.5)	2 (0.4)	
**Sex**			<.001
Male	2012 (42.2)	234 (51.7)	
Female	2756 (57.8)	219 (48.3)	
**Age at COVID-19 diagnosis (years)**			<.001
<50	858 (18.0)	44 (9.7)	
50-59	987 (20.7)	57 (12.6)	
60-69	1365 (28.6)	104 (23.0)	
≥70	1558 (32.7)	248 (54.7)	
**AREA-LEVEL SOCIAL DETERMINANTS OF HEALTH**			
**Census region of treatment oncology practice**			<.001
West	413 (8.7)	33 (7.3)	
Midwest	1437 (30.1)	121 (26.7)	
Northeast	620 (13.0)	136 (30.0)	
South	2293 (48.1)	163 (36.0)	
**Population density of treatment oncology practice**			.38
Urban	4507 (94.5)	433 (95.6)	
Rural City/Town	256 (5.4)	20 (4.4)	
**Census region of patient's primary residence[a]**			<.001
West	422 (8.9)	33 (7.3)	
Midwest	1422 (29.8)	121 (26.7)	
Northeast	617 (12.9)	134 (29.6)	
South	2305 (48.3)	165 (36.4)	
**Population density of patient's primary residence[a]**			.51
Urban	4091 (85.8)	394 (87.0)	
Rural City/Town	675 (14.2)	59 (13.0)	
**Median household income[a,b]**			.24
<$43,125	821 (17.2)	75 (16.6)	
$43,125 - $54,047	1046 (21.9)	91 (20.1)	
$54,048 - $68,446	1177 (24.7)	108 (23.8)	
>$68,446	1403 (29.4)	156 (34.4)	
**Percentage of population with only a high school diploma[a,b]**			.04
<25.5%	1501 (31.5)	119 (26.3)	
25.5% - 33.7%	1604 (33.6)	160 (35.3)	
33.8% - 41.2%	1067 (22.4)	117 (25.8)	

© 2023 Llanos AAM et al. *JAMA Network Open*.

>41.2%	279 (5.9)	35 (7.7)	
**Percentage of population (≤64 yrs.) with no health insurance**[a,b]			.001
<4.8%	639 (13.4)	77 (17.0)	
4.8% - 8.8%	1312 (27.5)	152 (33.6)	
8.9% - 14.7%	1472 (30.9)	129 (28.5)	
≥14.8%	1028 (21.6)	73 (16.1)	
**Percentage of population reporting White race**[a,b]			<.001
≤77.3%	1766 (37.0)	221 (48.8)	
77.4% - 92.1%	1845 (38.7)	144 (31.8)	
92.2% - 97.4%	683 (14.3)	53 (11.7)	
≥97.5%	146 (3.1)	12 (2.6)	
**Percentage of population reporting Hispanic ethnicity**[a,b]			.40
<0.7%	109 (2.3)	10 (2.2)	
0.7% - 3.1%	880 (18.5)	89 (19.6)	
3.2% - 9.5%	1771 (37.1)	154 (34.0)	
≥9.5%	1691 (35.5)	178 (39.3)	

Abbreviations: AAPI, Asian American and Pacific Islander; AIAN, American Indian and Alaska Native; NHB, non-Hispanic Black; NHW, non-Hispanic White.

[†] The Chi-Square test was used for comparisons of proportions across race and ethnicity categories.

[a] Social determinants of health variables were estimated based on census-level data for patient's primary area of residence at cancer diagnosis.

[b] To prevent reidentification of Registry patients by way of their residential area, the SDOH variables were segmented into quartiles.

© 2023 Llanos AAM et al. *JAMA Network Open*.

eFigure 1. Primary Reason for Delay or Discontinuation of Cancer Treatment Following a Confirmed Positive SARS-CoV-2 Test Result (n = 4768)

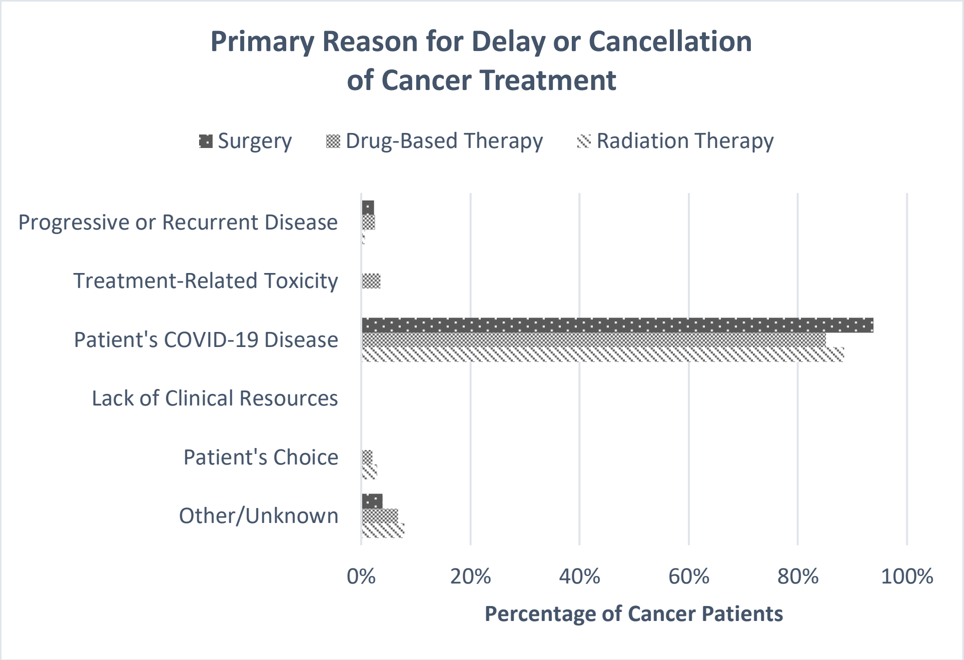

The primary reason for delay or discontinuation of all three cancer treatments was the patient's COVID-19 disease (surgery: N=120, 93.8%; pharmacotherapy: N=1151, 85.1%; and radiation therapy: N=123, 88.5%).

© 2023 Llanos AAM et al. *JAMA Network Open*.

eFigure 2. Relative Risk Regression Using a Poisson Distribution, Log Link, and Robust Error Variances

≥14-Day Delay or Discontinuation of ANY CANCER TREATMENT, N = 3212

Area-Level SDOH	RR (95% CI)	Log RR (95% CI)	*P* Value
**Census region**			
West	1.09 (0.95, 1.25)	0.09 (-0.05, 0.22)	.21
Midwest	0.82 (0.74, 0.91)	-0.19 (-0.30, -0.09)	<.001
Northeast	1.10 (0.98, 1.24)	0.10 (-0.02, 0.22)	.10
South	ref.	ref.	
**Population density**			
Urban	ref.	ref.	
Rural City/Town	0.92 (0.80, 1.06)	-0.08 (-0.22, 0.06)	.25
**Median household income**			
<$43,125	1.20 (1.02, 1.42)	0.19 (0.02, 0.35)	.03
$43,125 - $54,047	1.11 (0.97, 1.28)	0.11 (-0.03, 0.25)	.14
$54,048 - $68,446	1.00 (0.89, 1.13)	0.00 (-0.12, 0.13)	.94
>$68,446	ref.	ref.	
**% of pop with only high school diploma**			
<25.5%	1.26 (1.01, 1.57)	0.23 (0.01, 0.45)	.04
25.5% - 33.7%	1.17 (0.95, 1.43)	0.15 (-0.05, 0.36)	.13
33.8% - 41.2%	1.20 (0.98, 1.46)	0.18 (-0.02, 0.38)	.07
>41.2%	ref.	ref.	
**% of pop with no health insurance**			
<4.8%	1.25 (1.05, 1.49)	0.22 (0.05, 0.40)	.01
4.8% - 8.8%	1.24 (1.08, 1.42)	0.21 (0.08, 0.35)	.002
8.9% - 14.7%	1.07 (0.95, 1.20)	0.06 (-0.05, 0.18)	.29
≥14.8%	ref.	ref.	
**% of pop reporting White race**			
≤77.3%	ref.	ref.	
77.4% - 92.1%	0.87 (0.79, 0.95)	-0.14 (-0.23, -0.05)	.002
92.2% - 97.4%	0.93 (0.81, 1.05)	-0.08 (-0.21, 0.05)	.24
≥97.5%	1.08 (0.86, 1.36)	0.08 (-0.15, 0.31)	.50

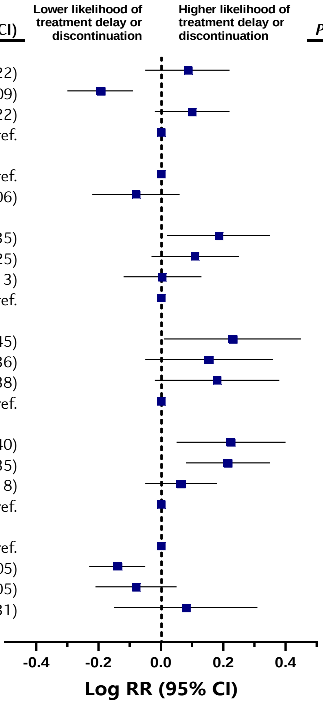

≥14-Day Delay or Discontinuation of SURGERY, N = 188

Area-Level SDOH	RR (95% CI)	Log RR (95% CI)	*P* Value
**Census region**			
West	0.88 (0.63, 1.23)	-0.13 (-0.47, 0.21)	.45
Midwest	0.99 (0.75, 1.31)	-0.01 (-0.29, 0.27)	.95
Northeast	0.73 (0.51, 1.03)	-0.32 (-0.67, 0.03)	.08
South	ref.	ref.	
**Population density**			
Urban	ref.	ref.	
Rural City/Town	1.17 (0.85, 1.61)	0.16 (-0.17, 0.48)	.34
**Median household income**			
<$43,125	1.14 (0.73, 1.78)	0.13 (-0.31, 0.58)	.56
$43,125 - $54,047	1.24 (0.86, 1.77)	0.21 (-0.15, 0.57)	.25
$54,048 - $68,446	1.16 (0.87, 1.55)	0.15 (-0.14, 0.44)	.31
>$68,446	ref.	ref.	
**% of pop with only high school diploma**			
<25.5%	1.76 (0.80, 3.89)	0.56 (-0.23, 1.36)	.16
25.5% - 33.7%	1.99 (0.91, 4.33)	0.69 (-0.09, 1.46)	.08
33.8% - 41.2%	1.32 (0.62, 2.80)	0.28 (-0.48, 1.03)	.47
>41.2%	ref.	ref.	
**% of pop with no health insurance**			
<4.8%	1.64 (1.08, 2.48)	0.49 (0.08, 0.91)	.02
4.8% - 8.8%	1.33 (0.94, 1.88)	0.28 (-0.07, 0.63)	.11
8.9% - 14.7%	1.00 (0.72, 1.38)	0.00 (-0.33, 0.32)	.99
≥14.8%	ref.	ref.	
**% of pop reporting White race**			
≤77.3%	ref.	ref.	
77.4% - 92.1%	0.88 (0.70, 1.11)	-0.12 (-0.35, 0.11)	.29
92.2% - 97.4%	0.94 (0.67, 1.32)	-0.06 (-0.40, 0.28)	.73
≥97.5%	1.54 (0.88, 2.69)	0.43 (-0.12, 0.99)	.13

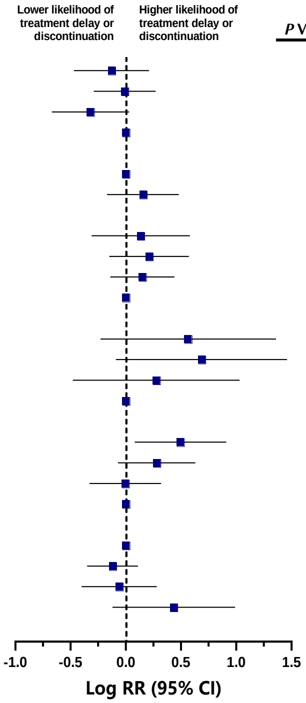

© 2023 Llanos AAM et al. *JAMA Network Open*.

≥14-Day Delay or Discontinuation of PHARMACOTHERAPY, N = 2920

Area-Level SDOH	RR (95% CI)	Log RR (95% CI)	*P* Value
**Census region**			
West	1.11 (0.96, 1.30)	0.11 (-0.04, 0.26)	.16
Midwest	0.85 (0.76, 0.95)	-0.17 (-0.28, -0.06)	.003
Northeast	1.16 (1.02, 1.32)	0.15 (0.02, 0.27)	.03
South	ref.	ref.	
**Population density**			
Urban	ref.	ref.	
Rural City/Town	0.88 (0.76, 1.03)	-0.12 (-0.27, 0.03)	.11
**Median household income**			
<$43,125	1.23 (1.03, 1.47)	0.21 (0.03, 0.38)	.02
$43,125 - $54,047	1.13 (0.97, 1.32)	0.12 (-0.03, 0.28)	.11
$54,048 - $68,446	1.01 (0.88, 1.15)	0.01 (-0.12, 0.14)	.91
>$68,446	ref.	ref.	
**% of pop with only high school diploma**			
<25.5%	1.30 (1.03, 1.65)	0.26 (0.03, 0.50)	.03
25.5% - 33.7%	1.18 (0.95, 1.46)	0.16 (-0.05, 0.38)	.14
33.8% - 41.2%	1.23 (1.00, 1.53)	0.21 (0.00, 0.42)	.06
>41.2%	ref.	ref.	
**% of pop with no health insurance**			
<4.8%	1.23 (1.01, 1.49)	0.20 (0.01, 0.40)	.04
4.8% - 8.8%	1.22 (1.06, 1.42)	0.20 (0.05, 0.35)	.007
8.9% - 14.7%	1.07 (0.95, 1.22)	0.07 (-0.06, 0.20)	.27
≥14.8%	ref.	ref.	
**% of pop reporting White race**			
≤77.3%	ref.	ref.	
77.4% - 92.1%	0.87 (0.78, 0.96)	-0.14 (-0.24, -0.05)	.004
92.2% - 97.4%	0.94 (0.82, 1.08)	-0.06 (-0.20, 0.08)	.38
≥97.5%	1.10 (0.86, 1.40)	0.10 (-0.15, 0.34)	.43

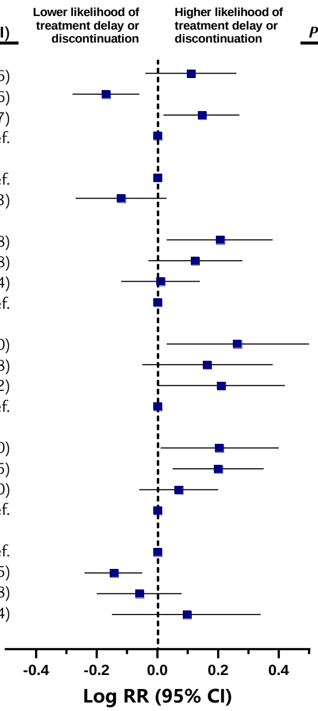

≥14-Day Delay or Discontinuation of RADIOTHERAPY, N = 300

Area-Level SDOH	RR (95% CI)	Log RR (95% CI)	*P* Value
**Census region**			
West	0.94 (0.54, 1.65)	-0.06 (-0.62, 0.50)	.83
Midwest	0.98 (0.69, 1.38)	-0.02 (-0.37, 0.32)	.89
Northeast	1.13 (0.75, 1.69)	0.12 (-0.28, 0.53)	.56
South	ref.	ref.	
**Population density**			
Urban	ref.	ref.	
Rural City/Town	1.00 (0.66, 1.52)	0.00 (-0.41, 0.42)	.99
**Median household income**			
<$43,125	1.16 (0.63, 2.12)	0.15 (-0.46, 0.75)	.64
$43,125 - $54,047	0.98 (0.56, 1.71)	-0.02 (-0.58, 0.54)	.94
$54,048 - $68,446	1.01 (0.62, 1.65)	0.01 (-0.48, 0.50)	.97
>$68,446	ref.	ref.	
**% of pop with only high school diploma**			
<25.5%	1.09 (0.50, 2.35)	0.08 (-0.69, 0.85)	.83
25.5% - 33.7%	1.17 (0.59, 2.32)	0.16 (-0.52, 0.84)	.65
33.8% - 41.2%	1.47 (0.77, 2.84)	0.39 (-0.27, 1.04)	.25
>41.2%	ref.	ref.	
**% of pop with no health insurance**			
<4.8%	1.02 (0.53, 1.97)	0.02 (-0.64, 0.68)	.96
4.8% - 8.8%	1.10 (0.71, 1.71)	0.10 (-0.35, 0.54)	.67
8.9% - 14.7%	1.04 (0.71, 1.53)	0.04 (-0.35, 0.43)	.84
≥14.8%	ref.	ref.	
**% of pop reporting White race**			
≤77.3%	ref.	ref.	
77.4% - 92.1%	1.06 (0.78, 1.43)	0.06 (-0.25, 0.36)	.72
92.2% - 97.4%	0.89 (0.54, 1.47)	-0.11 (-0.61, 0.39)	.66
≥97.5%	1.27 (0.61, 2.66)	0.24 (-0.50, 0.98)	.53

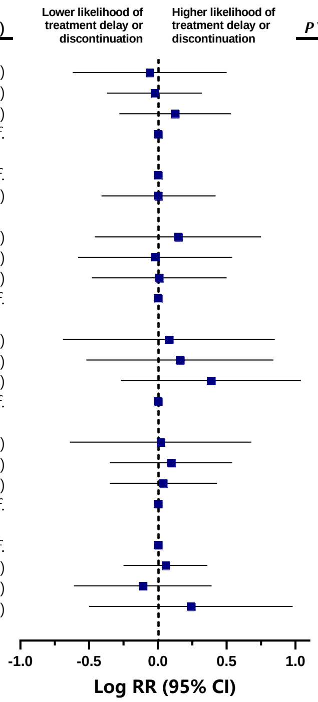

RRs examined the association between area-level social SDOH factors in the patient's area of residence with receipt of delayed or discontinued cancer treatment (as opposed to on schedule or timely cancer treatment), *following a confirmed SARS-Cov-2 test*. Area-level SDOH factors included census region, population density, median household income, percent of population with only a high school diploma, percent of population (≤64 years) with no health insurance, and percent of population reporting White race. Cancer treatment outcomes included delay/discontinuation of surgery, pharmacotherapy, radiotherapy, and any cancer treatment. All RRs were insignificant between area-level SDOH and receipt of delayed or discontinued surgery, pharmacotherapy, and radiotherapy. However, patients residing in census tracts in the Midwest vs. South were 18% less likely to experience delay/discontinuation of any cancer treatment (RR 0.82, 95% CI: 0.74-0.91; *P*<.001).

© 2023 Llanos AAM et al. *JAMA Network Open*.

eFigure 3. Adjusted Survival Curves of Time to Restart Pharmacotherapy Following a Confirmed Positive SARS-CoV-2 Test Result, Stratified by Race and Ethnicity, Separately for Men (n = 351) and Women (n = 506)

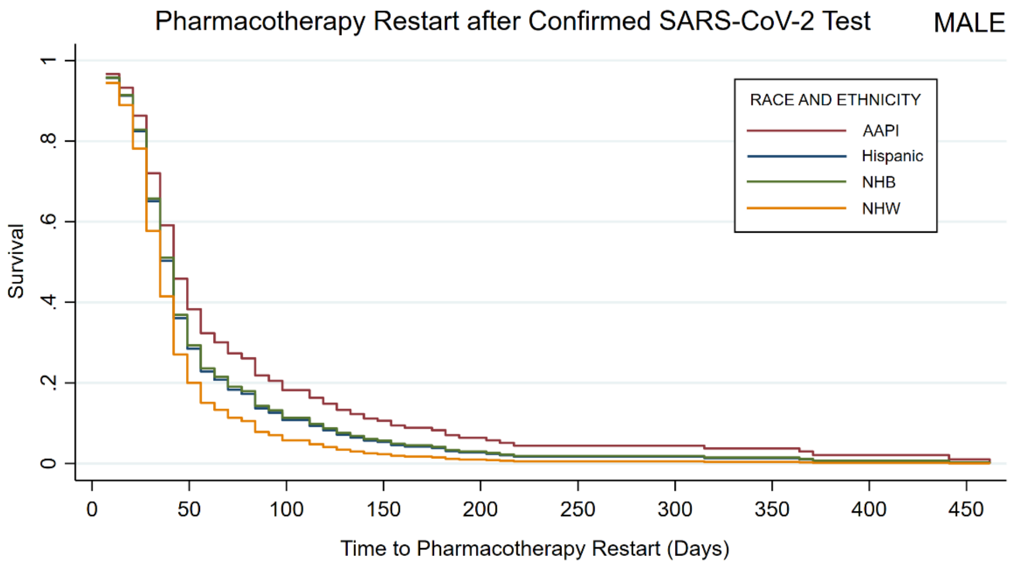

No. at risk										
AAPI	13	3	2	1	1	1	1	1	1	1
Hispanic	42	11	3	1	0	0	0	0	0	0
NHB	32	10	5	2	0	0	0	0	0	0
NHW	264	57	20	11	7	4	4	3	1	0

© 2023 Llanos AAM et al. *JAMA Network Open*.

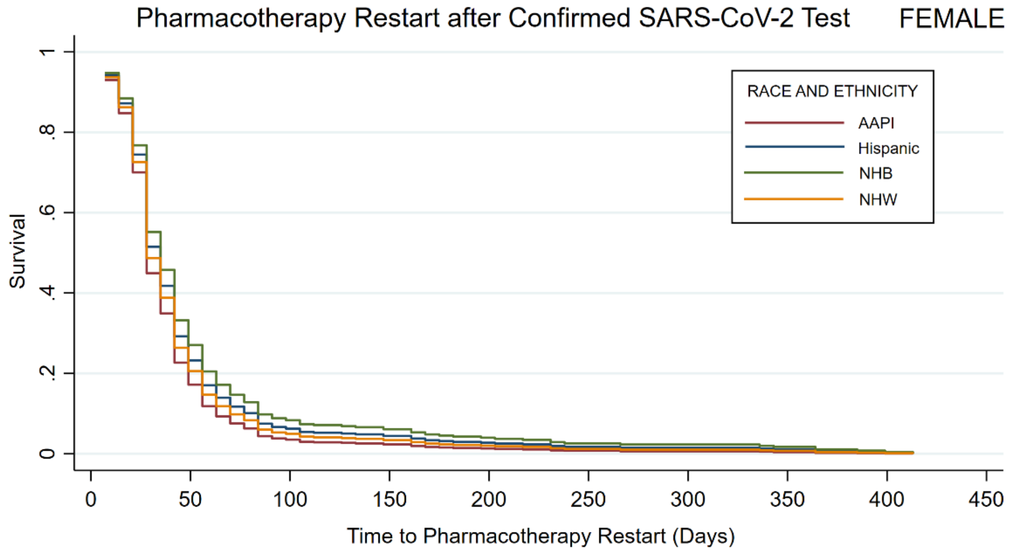

**No. at risk**									
AAPI	12	3	1	0	0	0	0	0	0
Hispanic	61	18	3	3	1	0	0	0	0
NHB	82	25	4	3	3	2	1	1	0
NHW	351	67	22	15	9	6	6	4	1

A Cox regression analysis was performed to assess differences in the restart rate for pharmacotherapy (first or only anti-cancer drug-based treatment) by race and ethnicity, separately for males and females, while adjusting for age and cancer type. Significant differences in pharmacotherapy restart rate across race and ethnicity categories were not observed, separately for males and females. There were also no trends in the effect estimates for females (all HRs approximated null). For males, while not statistically significant, compared to non-Hispanic White (NHW) cancer patients, the restart rate for pharmacotherapy was 24% less (HR, 0.76 [95% CI, 0.58-1.01]; *P*=.06) for non-Hispanic Black (NHB) patients, 22% less (HR, 0.78 [95% CI, 0.58-1.06]; *P*=.11) for Hispanic patients, and 40% less (HR, 0.60 [95% CI, 0.27-1.33]; *P*=.21) for non-Hispanic Asian American and Pacific Islander (AAPI) patients, while accounting for age and cancer type.

© 2023 Llanos AAM et al. *JAMA Network Open*.

eFigure 4. Adjusted Survival Curves of Time to Restart Pharmacotherapy Following a Confirmed Positive SARS-CoV-2 Test Result, Stratified by Age at COVID-19 Diagnosis (n = 857)

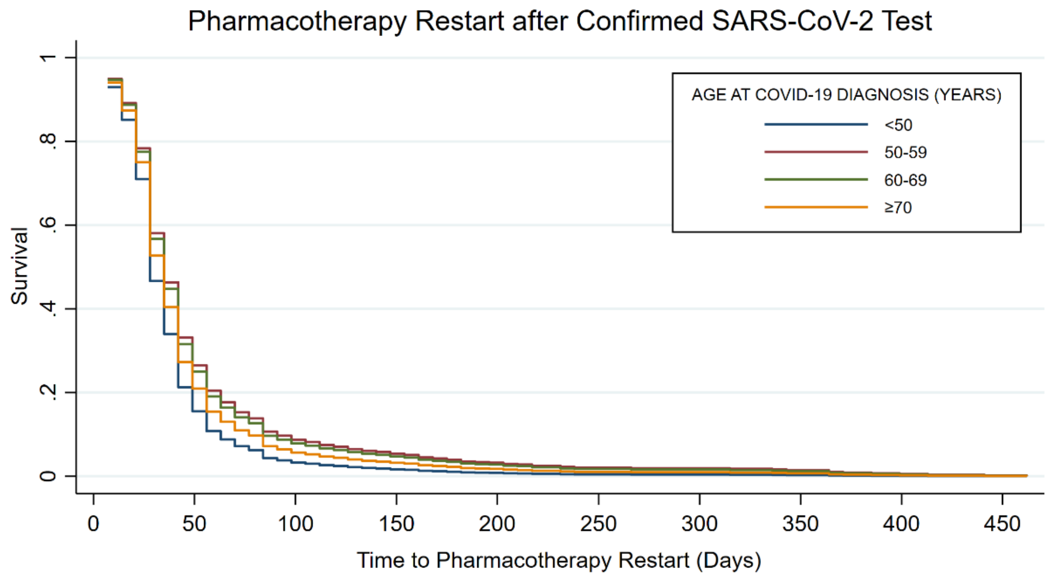

No. at risk										
<50 years	168	25	7	4	2	2	2	1	0	0
50-59 years	187	50	16	9	6	4	4	3	1	0
60-69 years	254	66	23	13	6	4	3	2	1	0
≥70 years	248	53	14	10	7	3	3	3	1	1

A Cox regression analysis was performed to assess differences in the restart rate for pharmacotherapy (first or only anti-cancer drug-based treatment) by age group at COVID-19 diagnosis, while adjusting for race and ethnicity and cancer type. Compared to cancer patients aged <50 years, the restart rate for pharmacotherapy was 29% less (HR, 0.71 [95% CI, 0.57-0.88]; *P*=.002) among patients aged 50-59 years and 26% less (HR, 0.74 [95% CI, 0.60-0.92]; *P*=.006) among patients aged 60-69 years, while accounting for race and ethnicity and cancer type. There was no significant difference in the pharmacotherapy restart rate between patients aged <50 years versus ≥70 years (*P*=.12).

© 2023 Llanos AAM et al. *JAMA Network Open*.

eFigure 5. Adjusted Survival Curves of Time to Restart Pharmacotherapy *Following a Confirmed Positive SARS-CoV-2 Test Result,* Stratified by Cancer Type (n = 857)

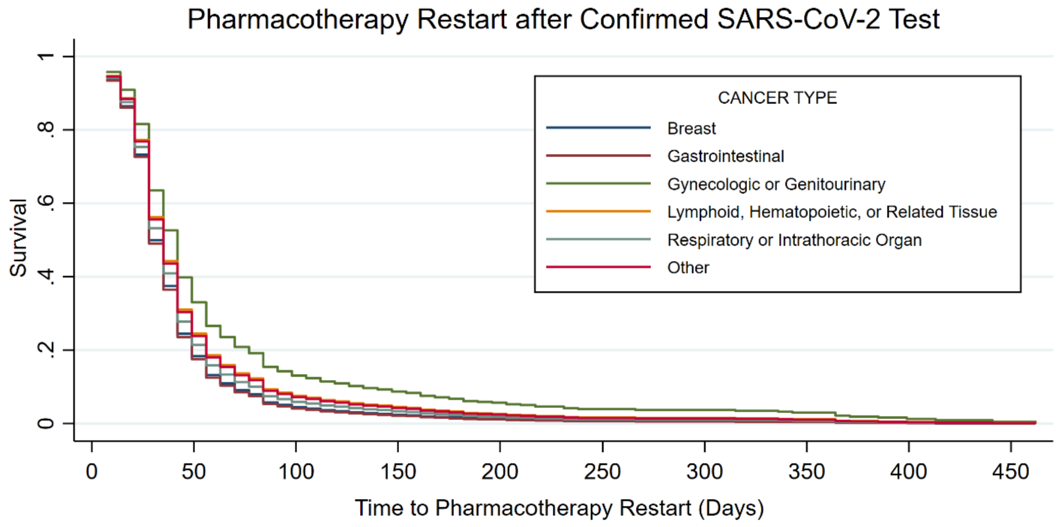

No. at risk										
Breast	233	46	13	8	6	4	3	3	1	0
Gastrointestinal	143	22	7	4	2	0	0	0	0	0
Gynecologic or Genitourinary	104	38	14	10	6	3	3	1	1	0
Lymphoid, Hematopoietic, or Related Tissue	203	55	18	9	4	3	3	2	1	1
Respiratory or Intrathoracic Organ	91	15	3	3	1	1	1	1	0	0
Other	83	18	5	2	2	2	2	2	0	0

A Cox regression analysis was performed to assess differences in the restart rate for pharmacotherapy (first or only anti-cancer drug-based treatment) by cancer type, while adjusting for race and ethnicity and age. Compared to patients with breast cancer, the restart rate for pharmacotherapy was 35% less (HR, 0.65 [95% CI, 0.51-0.83]; *P*=.001) among patients with gynecologic or genitourinary cancers, while accounting for race and ethnicity and age. However, there were no significant differences in pharmacotherapy restart rate between patients with breast cancer and all the other (i.e., gastrointestinal, lymphoid, hematopoietic, or related tissue, respiratory or intrathoracic organ) cancers (*P*>.05).

© 2023 Llanos AAM et al. *JAMA Network Open*.
