# Supplementary material for: Evaluation of Inequities in Cancer Treatment Delay or Discontinuation Following SARS-CoV-2 Infection
Source: JAMA Netw Open. 2023 Jan 13;6(1):e2251165. doi: 10.1001/jamanetworkopen.2022.51165 (PMC9856904; doi:10.1001/jamanetworkopen.2022.51165)
Supplement: Supplement 2. — Data Sharing Statement [file jamanetwopen-e2251165-s002.pdf]

Data Sharing Statement

Llanos. Evaluation of Inequities in Cancer Treatment Delay or Discontinuation Following SARS-CoV-2 Infection. *JAMA Netw Open*. Published January 13, 2023. doi:10.1001/jamanetworkopen.2022.51165

Data
Data available: No

Additional Information
Explanation for why data not available: The authors do not own the rights to the ASCO Registry data. However, the data may be available to others by request to the American Society of Clinical Oncology, Inc.
